# Emergency department visits at Rambam health care campus, Israel: non-trauma related dental conditions

**DOI:** 10.1186/s13584-020-00385-2

**Published:** 2020-05-22

**Authors:** Leon Bilder, Jacob Horwitz, Hadar Zigdon-Giladi, Zvi Gutmacher

**Affiliations:** 1 Department of Periodontology, School of Graduate Dentistry, Rambam Health Care Campus, Haifa, Israel; 2 Department of Maxillofacial Rehabilitation and Temporomandibular Disorders Unit, School of Graduate Dentistry, Rambam Health Care Campus, Haifa, Israel

**Keywords:** Non-traumatic dental conditions, Emergency department, Oral and maxillofacial surgery department

## Abstract

**Objectives:**

Studies of emergency department (ED) visits for non-traumatic dental conditions (NTDCs) have been carried out in the USA and Canada. In Israel, there is a shortage of such studies. In the current retrospective study, we report on the frequency and distribution of NTDCs ED visits at Rambam Health Care Campus (Rambam), in Haifa, which is an academic hospital serving more than 2.4 million residents of Northern Israel.

**Materials and methods:**

The data concerning ED visits at Rambam between 2010 and 2017 were obtained retrospectively from Rambam’s computerized clinical and personal database of adult patients (≥18 years) visiting the ED for NTDCs.

**Results:**

Overall, 1.8% of the patients who visited the Rambam ED, were identified as presenting with NTDCs. From 2010 until 2017, the number of NTDCs admissions increased by 45%, while the total ED admissions rose by 16%. The average waiting time for maxillofacial consultations for patients with NTDCs increased from 102 min in 2010 to 138 min in 2017. The busiest hours in the ED for NTDCs were during the morning shifts (47% of daily visits).

**Conclusions:**

The results of the study show that systemic and conceptual changes are needed to reduce the number of non-trauma related applications to ED.These changes can be by increasing the number of personnel or by introducing recent advances such as tele-medicine for prescreening of patients. This change calls for a greater involvement of the health policy leaders to provide alternative solutions for emergency dental care.

## Background

Oral health is one of the most important components of general health and contributes greatly to the quality of life [1]. According to the World Health Organization, oral diseases constitute a major burden on the public health in high-income countries, and a growing burden in many low-and middle-income countries [1]. As a consequence, individuals who are not able to maintain a good oral health, and fail to visit the dentist on a regular basis, may suffer oral complications which may require emergency treatment [2, 3]. Oral health neglect may lead patients to seek care at hospital emergency departments [4]. The emergency department (ED) is the gate of the hospital, through which enter non-elective patients [5]. Unfortunately, the emergency departments are generally not sufficiently staffed and equipped in order to provide adequate dental care, and most of the patients in need for urgent dental treatment receive only temporary palliative care, like analgesics and antibiotics [6]. Several studies conducted in the USA, concluded that most of the ED visits associated with oral health problems, could have been treated in dental offices in the community, or in hospital oral health departments [7, 8]. Studies from Canada showed a high number of repeated visits to the ED, suggesting that ED settings are ineffective in addressing dental problems [9]. According to the analysis of Sun et al., 2015 [10], visits to the ED for tooth-related pain and disease constitute a significant part of the public health budget, and therefore, in order to reduce the number of such visits, greater efforts should be invested in providing interventions at multiple levels [10]. We are aware of the Patient-Centered Medical-Dental Home (PCM-DH) project, which aspires to build an enhanced dental health care model in the United States. According to this model, each patient has a personal physician and/or dentist who leads a team of clinical care providers and staff who take collective responsibility and take care of all patient’s health care needs [11]. Implementation of such a model in Israel might reduce the number of visits to the ED, but unfortunately this is unlikely to happen in the near future.

To the best of our knowledge, the current study is the first to consider ED visits for non-trauma related dental conditions (NTDCs) in Israel. We retrospectively extracted the medical files of patients who had visited the ED at Rambam Health Care Campus (Rambam). Rambam is the largest academic hospital in northern Israel, serving a population of more than 2.4 million people (approximately 28% of the Israeli population), and is a tertiary referral center for twelve district hospitals.

The aim of the current study is to report on the frequency, and distribution of ED visits for NTDCs at Rambam, during the period from January 2010 to December 2017.

## Materials and methods

Rambam is a 1000-bed teaching hospital, comprising 45 medical units, with approximately 75,000 patients hospitalized annually. The main Emergency Department at Rambam has 40 beds and provides treatment to an average of 251 patients per day [5].

The data for the number of visits at Rambam’s ED between January 1, 2010 and December 31, 2017 were obtained from Rambam’s computerized database. Data was extracted according to the following inclusion criteria: Age ≥ 18 years old; NTDCs; Oral/Maxillofacial consultation received. Exclusion criteria: ED visit for a trauma related dental condition (TRDC). The data included: age, gender, residence, nationality, date of the visit, time spent in the ED, time for Oral/Maxillofacial consultation, self-reported main complaints, and diagnosis.

Statistical analysis was conducted using the SPSS 25 software package (SPSS Inc., Chicago, IL). Comparisons were performed using one-way ANOVA or Kruskal-Wallis when appropriate, for quantitative parameters. Chi-square tests were used for categorical parameters. Spearman’s rank correlation coefficient was used to check for monotonic relationships. *P* < 0.05 was considered as significant.

## Results

Between the years 2010–2017, 747,143 adult patients were admitted to Rambam’s ED. Among them, 3.1% (23,076 patients) were examined by a dentist from the Oral and Maxillofacial Surgery Department (OMSD). The dental diagnoses were classified into two categories: TRDCs which occurred in 42.6% of the patients, and NTDCs which occurred in 57.4% of the patients. Therefore, 1.8% of the patients admitted to Rambam ED, were identified as having NTDCs.

**Fig. 1:**
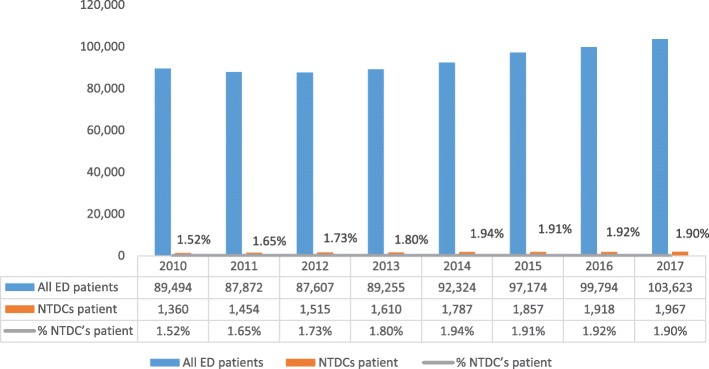
All patient visits in the ED, NTDCs patient visits in the ED, and percent of NTDCs patient visits in the ED

**Fig. 2:**
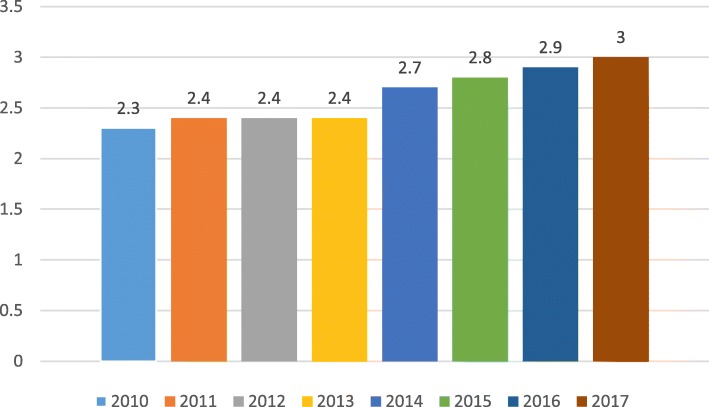
The average length of stay of NTDCs in the ED by year (hours)

Between 2010 and 2017, the number of ED admissions increased by 15.8% (89,494 and 103,623, respectively). During this period of time, the number of NTDCs admissions increased by 44.6% (1360 and 1967 respectively; see Fig. 1). During the same period of time, the average time spent by the NTDCs patients in the ED, increased from 2.3 to 3 h (30.4%, Fig. 2).

The patient median waiting time for consultation by OMSD staff members increased from 102 min in 2010, to 138 min in 2017 (35.2%, Table 1).

**Table 1:** Waiting time for consultation by OMSD staff (in minutes)

Year	N	Mean	Median	Std. Deviation
2010	1360	101.8	58.2	138.8
2011	1454	99.4	64.8	122.0
2012	1514	99.9	61.8	116.3
2013	1610	100.2	66.0	116.0
2014	1787	114.5	73.2	130.7
2015	1857	113.8	75.0	134.5
2016	1918	123.4	76.8	141.3
2017	1967	137.8	97.2	141.4
Total	13,467	112.8	72.0	131.8

**Fig. 3:**
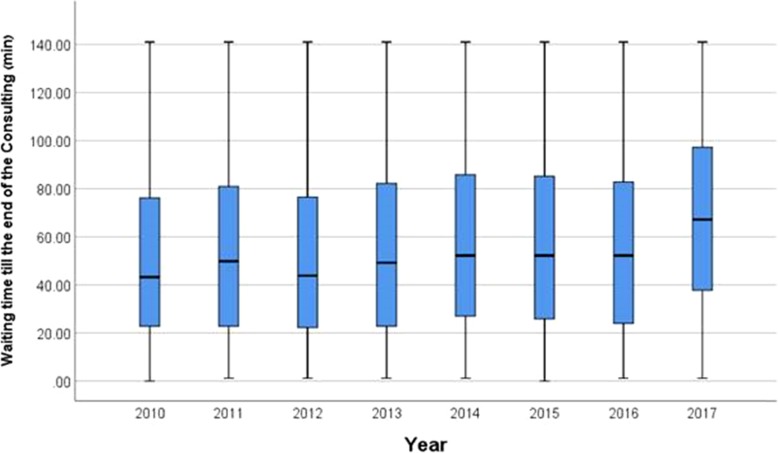
Percentiles (25–75) of waiting time for patients with NTDCs for consultation

The results show a correlation between the median waiting time for consultation and the passing of years (r = 0.109, *p* < 0.0001) (Fig. 3).

**Fig. 4:**
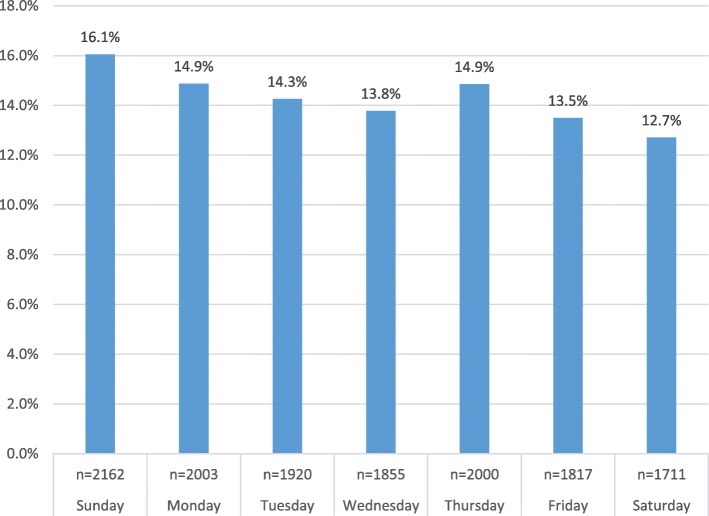
Distribution for which day of the week patients visit the hospital ED

The peak hours in the ED were during the morning and evening shifts (46.6 and 39% of NTDCs) and were equally distributed during the week, with a slightly higher frequency on Sundays (the first day of the workweek in Israel). Saturday (Israeli weekend) was a less busy day with a 12.7% frequency of visits to ED. In contrast, on Sundays the percentage of overall NTDCs visits to the ED was 16.1% (Fig. 4).

The mean age of the NTDC patients was 38.9 ± 20.8 years, with similar gender distribution (female 49.1%, male 50.1%). 93.7% of patients were of northern Israel, North and Haifa districts, and 52.2% were Jews.

## Discussion

In the current study, 3.1% of ED visits between 2010 and 2017 were examined by the OMSD, of which 58% were diagnosed as NTDCs. Similar results were reported in other countries: In the USA, during the years 2007 to 2010, 1.7% of ED visits were reported as NTDC [6]. Similarly, in 2010, the Centers for Disease Control and Prevention reported that 1.5% of ED visits were NTDCs [12]. Surveys in Oregon, Missouri, Florida and Iowa found 2.5% [10], 1.7% [13], 1.4 [14], 2.1% [15] and 1.5% [16], respectively, of ED visits were NTDCs. A report from Alberta, Canada found that 1.2% of ED visits were NTDCs [4].

Although the health systems in the US, Canada and Israel have differences related to organizational structure, funding, and attitude towards public health, the frequency that patients seek emergency dental health care in hospital settings seems to be quite similar.

There was a relative increase in dental visits as opposed to medical ED visits as demonstrated by an increase of 15.8% in Rambam’s ED visits between 2010 and 2017 while there was a 44.6% increase in NTDCs visits.

A similar trend was found in other studies. In a national study in the US, between 2006 and 2009, the incidence of ED visits for patients seeking dental treatment increased by 16%, rising from 874,000 to 936,432 visits [17].

State studies in the US show similar results: Hong et al., 2011 [13] showed a 45.6% increase in dental complaint visits during 2001–2006 in Kansas City, Missouri; Tomar et al., 2016 [14], found that NTDCs in Florida increased from 104,646 in 2005 to 163,906 visits in 2014, 56.6% [14]. Other studies from the US show similar trends [18–20].

NTDCs patients in 2017 spent 30.4% more time in ED than in 2010. Similarly, OMSD consultation time increased by 35.2%. This may reflect various factors, such as the increased volume in the ED, more serious pathology, less adequate OSND staffing or a change in priorities.

In the current study, an equal gender distribution of NTDCs was found. This result differs from other studies which found a higher percentage of female visits [6, 13–15].

Israel’s population (especially in the northern area) consists of different ethnic and religious groups. The Northern district, and the Haifa district within the district, is comprised of 43 and 68% Jews, respectively. In the present study, 51.4% of the ED patients and 52.2% of NTDCs patients were Jewish, respectively. In the current study we did not find significant statistical differences between NTDC ED services when analyzing population (Jewish\Arab), or gender (female\male).

The mean patient age was 38.9 ± 20.8, the age group of 18–50 years being the largest sub-set (68.7%) of the NTDC group. Persons aged 25–34 years constituted the largest group according to Tomar et al., 2016 [14], 20–44 years old group was the main group treated in the EDs of Alberta, Canada [4], and Meyer et al., 2016 [21] found that 62% of patients with dental-related ED visits were 18–44 years old.

Regarding the distribution among the week, Saturday (the Jewish day of rest), was a less busy day, with 12.7% of the weekly NTDCs patients. Sunday (the first workday in Israel) was busier with 16.1% NTDCs. The present study corroborates the study of Whitt W. & Zhang X. 2017 [22] at Rambam, and found Sunday to be the busiest day in ED, with a steady decrease during the course of the week. In contrast, Hong et al., 2011 [13], Serna et al., 2017 [15], and Meyer et al., 2016 [21] reported that Sunday (referring to the day of rest in most countries) had the highest load of NTDCs patients (16.9% of ED admittances). This *phenomenon* can be explained by differences in social, religious and behavioral factors. In addition, Israel provides paid sick days for short and/or long-term illnesses to the majority of its employees. In comparison, many U.S. workers have no paid sick days, protected leave is provided only to certain eligible employees, and unpaid leave for serious illnesses [23]. As a result, the NTDCs patients prefer to turn to ED on their free days.

The morning shift was the busiest (46.6, 39.0 and 14.4%, for the morning, evening and night shifts, respectively), which closely resembles the distribution in Serna et al., 2017 [15], who also found the morning shift busier. According to Tomar et al., 2016 [14], the distribution of arrival times for NTDCs visits were lowest at 5 AM, increased sharply after 6 AM, peaked at 10–11 AM, declined slightly through the afternoon hours, peaked again at 6 PM, and then dropped off during the late-night hours.

In Israel, there is universal insurance coverage, which is guaranteed by the 1995 National Health Insurance (NHIL) law. Israel’s permanent residents can choose from four competing, nonprofit health plans [24]. For many years, dental care has not been included in the NHIL basket of services. Dental care for children was first introduced in 2010, initially up to 8 years of age. Only from 2019 did children from 0 to 18 years of age begin to receive free dental care. Also, from 2019, elderly people (over 75 years of age) are granted basic oral health care services. Since the current dental component of the NHIL includes children and the elderly, the largest group of adults, aged 19–74, are currently excluded. Dental treatment has been traditionally provided on an “out-of-pocket” expense basis, therefore, patients may try to seek “free” ED assistance before resorting to private options.

Dental related ED admissions constitute an ever-increasing burden on the already overloaded ED. In the current study, 93.7% of Rambam ED NTDC patients were from the Northern and Haifa districts. The number of the total population of these districts is more than 2.4 million, representing about 28% of Israel’s population [25]. While the northern population represents a large percentage of the Israeli population, other centers should be studied as well as, the populations differ and different kinds of care are offered locally.

The results of the study show that systemic and conceptual changes are needed to reduce the number of non-trauma related applications to ED. These changes can be by increasing the number of personnel or by introducing recent advances such as tele-medicine for prescreening of patients. Prescreening of patients can be provided by skilled general dentists working in emergency dental clinics who will perform a quick screening. The clinic would be operated by health maintenance organization. In addition, establishing online services for virtual primary consultation may lower the load in ED and reduce waiting time.

The study did not take into account socioeconomic variables, such as the patient’s level of education, occupation, employment status, and ethnicity. These factors may impact his/her perception of dental care and dental complaints. Furthermore, this distribution is different in the northern region as compared to other areas in Israel. Another limitation, is that records for the same person with multiple ED visits could not be linked. Therefore, information regarding whether a certain ED visit was an isolated event or a repeated visit, is missing.

## Conclusion

According to the study, 1.8% of the patients admitted to Rambam ED, were identified as having NTDCs. These results are similar to those in the US and Canada. The number of visits and their duration increased substantially during the past eight years. The NTDCs patient’s increase was much greater than those of general ED admissions. The results of this investigation show that the NTDC’s patients spend more and more time in ED which leads to a serious burden on ED. Health policy leaders should make greater efforts to provide alternative solutions other than ED for emergency dental care.

## Supplementary information

**Additional file 1.** Population by district, sub-district and religion.

## Data Availability

The datasets analyzed during the current study are available from the corresponding author on reasonable request. The datasets analyzed during the current study are available in Additional file 1 and from the corresponding author on reasonable request.

## Abbreviations

ED: Emergency department
NHIL: National Health Insurance Law
NTDC: Non-traumatic dental conditions
OMSD: Oral and Maxillofacial Surgery Department
Rambam: Rambam Health Care Campus
TRDC: Trauma related dental conditions
